# Design and Characterization of Model Systems that Promote and Disrupt Transparency of Vertebrate Crystallins In Vitro

**DOI:** 10.1002/advs.202303279

**Published:** 2023-10-28

**Authors:** Michael R. Bergman, Sophia A. Hernandez, Caitlin Deffler, Jingjie Yeo, Leila F. Deravi

**Affiliations:** ^1^ Department of Chemistry and Chemical Biology Northeastern University 360 Huntington Ave Boston MA 02115 USA; ^2^ Sibley School of Mechanical and Aerospace Engineering Cornell University 413 Upson Hall, 124 Hoy Rd Ithaca NY 14850 USA

**Keywords:** cataract, crowding, crystallins, hydrogel, optics

## Abstract

Positioned within the eye, the lens supports vision by transmitting and focusing light onto the retina. As an adaptive glassy material, the lens is constituted primarily by densely‐packed, polydisperse crystallin proteins that organize to resist aggregation and crystallization at high volume fractions, yet the details of how crystallins coordinate with one another to template and maintain this transparent microstructure remain unclear. The role of individual crystallin subtypes (α, β, and γ) and paired subtype compositions, including how they experience and resist crowding‐induced turbidity in solution, is explored using combinations of spectrophotometry, hard‐sphere simulations, and surface pressure measurements. After assaying crystallin combinations, β‐crystallins emerged as a principal component in all mixtures that enabled dense fluid‐like packing and short‐range order necessary for transparency. These findings helped inform the design of lens‐like hydrogel systems, which are used to monitor and manipulate the loss of transparency under different crowding conditions. When taken together, the findings illustrate the design and characterization of adaptive materials made from lens proteins that can be used to better understand mechanisms regulating transparency.

## Graphical abstract

Human vision is enabled through the transmission, refraction, and color‐sensing of light through the eye. Combining analytical and computational methods, the properties and interactions of lens crystallins that enable their organized, dense‐packing into a transparent microstructure are explored.

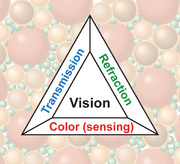

## 1 Introduction

Despite nearly two centuries of research on the vertebrate lens,^[^ ^1^, ^2^ ^]^ the molecular mechanisms underlying proper lens function, i.e., transparency, and ultimate dysfunction, i.e., cataractogenesis, are still not fully understood. Present at concentrations up to 400 mg mL^−1^,^[^ ^3^, ^4^, ^5^ ^]^ crystallins make up roughly 90% of the total soluble protein content in the lens which is delicately balanced by short‐range ordering via weak and non‐covalent interactions to maintain transparency.^[^ ^6^, ^7^, ^8^ ^]^ The human lens contains three conserved subtypes (α, β, and γ‐crystallin)^[^ ^9^, ^10^, ^11^ ^]^ that comprise 15 structural isoforms, with only βA1/A3 being products of alternative splicing. Except for γ‐crystallins, which remain monomeric, α and β‐crystallin form complex and heterogenous quaternary structures. The largest subtype, α‐crystallin, contributes to overall tissue transparency necessary for vision,^[^ ^8^, ^12^ ^]^ while the smallest subtype, γ‐crystallin, has the highest refractive index increment which contributes to efficient light refraction.^[^ ^13^, ^14^ ^]^ The intermediate subtype, β‐crystallin, is further segmented into trimers and dimers, referred to as β_L1_ and β_L2_ respectively, and larger oligomers (up to octamers confirmed in vitro), which are denoted as β_H_. The combination of these polydisperse structures is thought to facilitate dense protein packing through regulating osmotic pressure among other protective functions,^[^ ^12^, ^15^, ^16^, ^17^, ^18^, ^19^, ^20^, ^21^ ^]^ yet it is unclear how the adaptive polydispersity of β‐crystallins interplays with the molecular packing and coordination of the other crystallin subtypes.

Colloid and materials science informs us that polydispersity enables fluidity within densely packed environments, where an increase in the material volume fraction (ϕ) is often correlated to an increase in long‐range ordering of monodispersed systems.^[^ ^22^, ^23^, ^24^ ^]^ In the lens, crystallization is prevented at high protein volume fractions (larger ϕ) and instead short‐range order interactions are dominant.^[^ ^8^ ^]^ This is exemplified in the squid lens with the taxon‐specific S‐crystallin, where many isoforms of one subtype behave as patchy colloids that adopt multiple configurations to accommodate the difference in packing densities across the lens.^[^ ^25^ ^]^ Unlike the squid lens, the vertebrate lens must adopt separate mechanisms to achieve this function by coordinating three crystallin subtypes, with various oligomerization states that are expected to behave as hard spheres based on experimental evidence,^[^ ^16^, ^18^, ^26^ ^]^ and supported by theory.^[^ ^27^ ^]^ The maintenance of crystallin solubility under the crowded conditions of the vertebrate lens has puzzled researchers for decades. Are these products of excluded volume that are stabilized by electrostatics,^[^ ^28^ ^]^ or are specific interactions between crystallin subtypes regulating this process?

To investigate these questions, we designed and built an in vitro model of a crowded lens to first examine the explicit role of β‐crystallins in facilitating short‐range order for transparency. Using an integrated biophysical, chemical, and computational approach, we assayed crystallin mixtures that promote solubility under crowded conditions. Inelastic hard‐sphere simulations under increasing volume fractions were also used to further investigate the role of polydispersity and size of crystallin subtypes. This approach was paired with experimental surface pressure measurements to then approximate attractive and repulsive effects of specific crystallin pairings. Finally, we developed an in vitro lens model to mimic transparency loss due to crowding effects within a hydrogel matrix and evaluated potential means to delay these effects. Data gleaned from these studies highlight a significant role for β‐crystallins in regulating the cytosolic microstructure and transparency of lenticular fibers for vision.

## 2 Results and Discussion

### 2.1 Limits of Crystallin Solubility under Crowded Conditions

**Figure 1:**
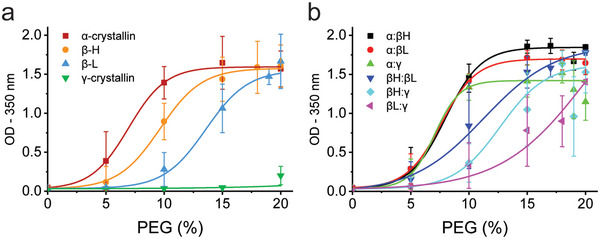
Molecular crowding impacts solubility of crystallin mixtures. Crystallins purified from bovine lens extract were mixed with increasing weight percentages of high‐molecular‐weight polyethylene glycol (PEG). Turbidity was measured as a function of optical density (OD) and curves were best fit to a Boltzmann equation. a) Isolated crystallin subtypes (1 mg mL^−1^) showed a size‐dependent correlation to resist crowding where α‐crystallin precipitated at the lowest PEG percentage and γ‐crystallin remained largely soluble even at 20% PEG. b) Mixtures of two subtypes at a 1:1 ratio were assayed to determine changes in solubility relative to individual subtypes. Results are listed in Table 1. Data points represent averages of biological replicates (*N* = 3) with error bars representing one standard deviation.

To investigate how short‐range order and transparency are maintained in the lens, we developed an assay to understand how molecular crowding affects the solubility of crystallins. We used polyethylene glycol (PEG, 35 000 Da) as a crowding agent to achieve high volume fractions of protein reminiscent of the lens environment. High molecular weight PEG has a greater effect on the system thermodynamics, promoting protein‐protein interactions through depletion forces in vitro.^[^ ^29^, ^30^, ^31^ ^]^ The utility of crowding agents, like PEG, to induce protein‐protein interactions and in some cases conformational changes, precipitation, or lipid membrane fusion in vitro have previously been reported in other systems,^[^ ^29^, ^30^, ^31^, ^32^, ^33^ ^]^ and has been used to study the solubility points of individual crystallins.^[^ ^34^ ^]^ To explore this, PEG was added at increasing weight percentages (w/v) to a starting solution of 1 mg mL^−1^ crystallin in PBS buffer, and changes in optical density (OD) were measured at 350 nm. We found that for individual crystallin subtypes, their respective limit of solubility under crowded conditions correlated with their size (**Figure** **1**a). For instance, 50% of maximal scattering (i.e., ½ solubility point) for α‐crystallin was estimated at 6.9% PEG, whereas γ‐crystallin remained almost completely soluble at even 20% PEG (**Table** **1**). The β‐crystallins had an intermediate solubility profile with 50% maximal scattering at 9.6% and 13.4% for the high and low sizes, respectively. These trends are in agreement with a previously published report.^[^ ^34^ ^]^

**Table 1:** Comparison of solubility between crystallin mixtures under crowded conditions. Using curve‐fitted data from Figure 1, the ½ solubility point in terms of weight percentage (PEG%) of crowding agent is reported. Dashes represent curves that did not reach a significant final plateau to assign solubility limits. The data for calf lens lysates containing all crystallins expectedly at native ratios (ALL) are reported as well. Data for these can be found in (Figure 1 and Figure S1, Supporting Information).

	½ Solubility [PEG%]		½ Solubility [PEG%]
α‐crystallin	6.9%	α:β_H_	7.9%
β_H_	9.6%	α:β_L_	7.5%
β_L_	13.4%	α:γ	7.0%
γ‐crystallin	–	β_H_:β_L_	11.0%
ALL (1 mg mL^‐1^)	8.0%	β_H_:γ	12.5%
ALL (10 mg mL^‐1^)	5.0%	β_L_:γ	–

Because the lens cytosol contains a heterogenous mixture of crystallins subtypes, we next explored how the ½ solubility point was affected when subtypes were assayed together. Protein solutions were prepared at a 1:1 mass ratio and kept at a standard mass concentration of 1 mg mL^−1^ to compare with the results of individual subtypes. Contrary to what we expected, γ‐crystallin did not increase the ½ solubility of α‐crystallin, but β‐crystallins did. Our data showed that β‐crystallins increased the ½ solubility point of a mixture with α‐crystallins from 6.9% PEG (α‐crystallin alone) to 7.5% and 7.9% for α:β_L_ and α:β_H_, respectively (Figure 1b). Furthermore, we noticed that the α:β mixtures had increased OD, saturating around 1.8 (A.U.) for α:β_H_ and 1.7 (A.U.) for α:β_L_ compared to 1.6 for individual subtypes indicating that the formed condensates had increased light scattering at a similar protein mass (Figure 1b). Prior evidence has shown that α‐crystallin and β_H_ often interact, even in other organisms.^[^ ^35^ ^]^ Another study supports that β‐crystallin isoforms can promote solubility in a protein mixture.^[^ ^36^ ^]^ This, along with our data, suggests that interactions between α‐ and β‐crystallins may be involved promoting transparency and consequently increasing turbidity when destabilized.

When all three crystallin subtypes were evaluated at their native mass ratios (35:37:21 α, β, γ) and at a 1 mg mL^−1^ total protein concentration, we observed a peak OD of around 1.6, similar to that of the individual subtypes, with an extrapolated ½ solubility point of ≈8% (Figure S1, Supporting Information). Because some pairings were more stable under crowded conditions, we suspect there are competing factors among native lens crystallins with some promoting and some compromising the overall solubility of the system. When assayed again at 10 mg mL^−1^ total protein concentration, the peak OD was 2.4 and the ½ solubility decreased to 5% PEG relative to the 1 mg mL^−1^ condition, suggesting that turbidity in a crowded solution is non‐linearly related to protein density.

Moreover, we observed a decrease in turbidity as the crowding conditions exceeded 15% PEG for some samples. Most notably in the crystallin mixtures with all three subtypes in their native ratio, at both 1 and 10 mg mL^−1^, turbidity decreased by 28.6 and 28.4% respectively between 15 and 20% PEG (Figure S1, Supporting Information). This phenomena was previously observed in the dilution of ox lens extracts, where a relatively transparent mixture became turbid after dilution, before clarifying through further dilution.^[^ ^37^ ^]^ This behavior may suggest a threshold in cooperativity between more complex mixtures of crystallins; beyond which, interactions may stabilize under extremely crowded conditions. Possible interactions include domain‐swapping or changes in oligomeric state under crowded conditions.^[^ ^38^, ^39^, ^40^ ^]^ However, due to material challenges associated with the viscosity and solubility of the PEG itself in our assay, we were not able to achieve higher densities of PEG to explore this further. Future work should further explore the changes in protein concentration or alternate ratios of lens crystallins in the mixture to interrogate possible mechanisms.

### 2.2 Hard‐Sphere Simulations Reveal Effects of β‐Crystallin on Packing

To understand relationships between volume fraction (ϕ), colloidal polydispersity, and long‐range order of crystallins in the lens, we used event‐driven molecular dynamics simulations (EDMD) of inelastic hard‐spheres to measure the changes in the radial distribution functions (RDF, denoted as *g*(**r**)) and static structure factors (S(**k**)) as ϕ increased from 0.1 (loosely packed) to 0.4 (densely packed). The radial distribution plots the likelihood of discovering a particle at a distance **r** away from a specific reference particle, while S(**k**) is the Fourier transform of *g*(**r**). The range of values of ϕ were motivated directly by the density of the human lens, which ranges from 250 to 400 mg mL^−1^ with corresponding volume fractions of ϕ = 0.2 to ϕ = 0.3.^[^ ^41^, ^42^ ^]^ Hard sphere simulations of α‐crystallins and β‐crystallins were previously used to describe the conditions needed to induce dynamical arrest, i.e., when particles effectively stop motion, observed in experimental studies.^[^ ^43^, ^44^, ^45^ ^]^ To build on this, we utilized the concentration ratios of crystallins that were previously determined in bovine lens^[^ ^46^ ^]^ along with average diameters measured with DLS (Figure S2, Supporting Information) to construct the particle compositions in the simulations.

**Figure 2:**
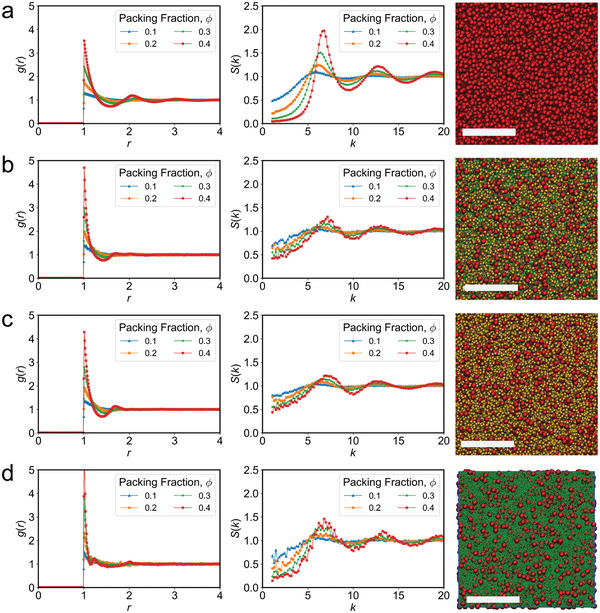
Event‐driven MD simulations of inelastic hard spheres reveal the effects of β‐ and γ‐crystallins on the crowding behavior of α‐crystallins as the volume fraction, ϕ, increased from 0.1 to 0.4. The first and second columns depict the radial distribution function (RDF) and the static structure factor (S(k)) in normalized units of length, σ. The third column illustrates each model system at ϕ = 0.1 with the corresponding scale bar length of 10 σ. a) Monodisperse α‐crystallin exhibited classical hard sphere behavior where the RDF and S(k) periodic peaks became increasingly prominent as ϕ increased. b) In polydisperse systems with α‐, β‐, and γ‐crystallins, both the RDF and S(k) of α‐crystallin had significantly diminished periodic peaks, signifying disrupted long‐range order, especially at low ϕ. c) Removing the γ‐crystallins resulted in the same behavior for α‐crystallins, while d) removing β‐crystallins instead had less of an effect on the long‐range order of α‐crystallin at the highest ϕ of 0.4.

Dense packing of monodisperse α‐crystallin resulted in characteristic periodic peaks appearing in both the RDF and S(**k**) (**Figure** **2**a), signifying periodic spacing and long‐range order as ϕ increased which is a fundamental characteristic of monodisperse hard spheres and disks when the system transitions from fluid‐like behavior to an arrested glassy state.^[^ ^43^, ^44^, ^47^, ^48^ ^]^ We suspect this long‐range ordering may decrease the transparency of the system, as it favors a more rigid structure with fewer short‐range interactions that are necessary for transparency. The characteristic periodic peaks in the RDF and S(**k**) of α‐crystallins were immediately diminished in a polydisperse system consisting of α‐, β‐, and γ‐crystallin particles represented with a mole fraction of 0.05:0.20:0.75 (α:β:γ), which is calculated from the experimental mass ratios,^[^ ^46^ ^]^ averaged across the lens and converted to moles using the estimated molecular weights based on known protein standards (BioRad) of 680, 180, and 21 kDa for α‐, β‐, and γ‐crystallin respectively (Figure 2b). In these simulations, β‐crystallin is more representative of β_H_, which is the most prominent oligomeric form of β‐crystallin in the lens.^[^ ^46^ ^]^ Moreover, there were leftward shifts in both the troughs and peaks of the RDF at ϕ = 0.4 compared to the regular periodicity in monodisperse α‐crystallins, where the peaks appeared at regular intervals of *r* = 1, 2, and 3. Polydispersity diminished the long‐range order present in the α‐crystallin only system. The largest frequency peaks in the S(**k**) at ϕ = 0.4 correspond to a wavelength of ≈18 nm after conversion from unitless to nm, agreeing well with previous ex vivo results of bovine lens.^[^ ^41^ ^]^ The observed RDF and S(**k**) for β‐crystallins (β‐β) in the polydisperse system showed a peak at ≈10 *k* and troughs at ≈6 and 12 *k* which inversely correlated to the peaks and troughs of α‐crystallin in a polydisperse system (Figure 2b; Figure S3). This implies that β‐crystallins could be distributed to surround α‐crystallin particles in crowded environments. In contrast, the S(**k**) of γ‐crystallins (γ‐γ) was essentially flat at all ϕ (Figure S3, Supporting Information), implying that the smallest particles may not directly contribute to ordering even at the highest volume fractions. Therefore, γ‐crystallins likely display fluid‐like behavior even when surrounded by the much larger α‐ and β‐crystallins.

The effects of polydispersity in lens crystallins were previously investigated in the context of α and γ‐crystallin mixtures.^[^ ^41^ ^]^ Inspired by these initial studies, we investigated the structural effects of β‐ and γ‐crystallins in a polydisperse system, where we selectively excluded either γ‐crystallins (Figure 2c) or β‐crystallins (Figure 2d) while retaining the same overall mass despite the increasing volume fraction. Omission of γ‐crystallins only produced a profile similar to the original polydisperse system, i.e., α:β:γ, which appeared dominated by short‐range ordering (Figure 2b,c). In contrast, when β‐crystallins were excluded, the RDF peak at *r* = 2 *σ* was maintained and the S(**k**) intensity depicted long‐range ordering of α‐crystallins, more similar to the monodispersed condition (Figure 2a,d). These contrasting changes in RDF and S(**k**) between the α:β and α:γ systems indicated differing roles where β‐crystallins strongly influenced the packing geometry of α‐crystallins, disrupting long‐range ordering. We also observed that the ratios of α:β:γ crystallins greatly impacted the degree of order. Systems with equal ratios (1:1:1) showed RDF and S(**k**) variations that were more similar to monodisperse conditions with decreased intensity in *g(r)* (Figures S4 and S5, Supporting Information). Together these data highlight an important contribution for both particle sizes and their distribution in maintaining short‐range order of α‐crystallins.

### 2.3 Measurement of Attractive and Repulsive Crystallin Interactions

**Figure 3:**
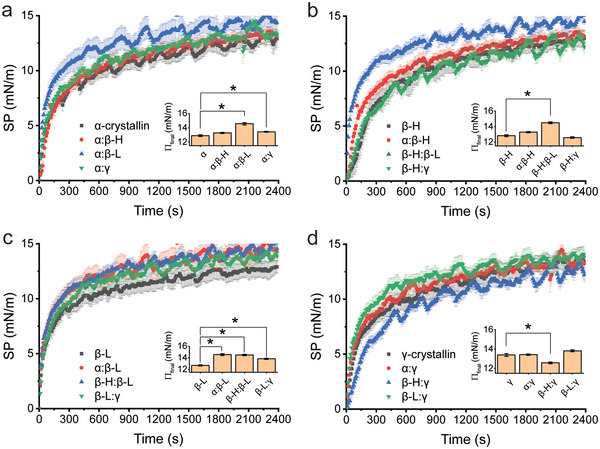
Surface pressure profiles of crystallin mixtures. Crystallin subtype mixtures at a 1:1 mass ratio compared to their relative control (gray squares) of a) α‐crystallin, b) β_H_, c) β_L_, or d) γ‐crystallin. Insets are statistical plots of final surface pressure (Π_final_) with asterisks signifying statistical significance compared to the control. Data are averaged from biological replicates (*N* = 2) with error bars reporting standard error of the mean.

We next investigated the role of chemical interactions on structure formation, where we monitored surface pressure (i.e., the change in surface tension with respect to time) of crystallin mixtures. Although this technique has been used to measure properties including aggregation potential of individual proteins^[^ ^49^, ^50^ ^]^ and antibody‐ligand interactions,^[^ ^51^ ^]^ this is the first report to our knowledge that uses surface tensiometry to monitor protein‐protein interactions. To do so, we first measured the surface pressure of each crystallin subtype (α, β_H_, β_L_, and γ) at 1 mg mL^−1^ over 40 min which established a reference point for each subtype. We next assayed subtype pairs mixed at a 1:1 mass ratio, maintaining the same total protein concentration of 1 mg mL^−1^, and monitored surface pressure. Both the initial rate of change in surface tension and the final achieved surface pressure were recorded (**Figure** **3**).

Initial rates and final surface pressure values for each individual subtype were similar ranging between 3.20–4.02 mN m^−1^ min^−1^, except for β_H_ which was significantly lower at 1.32 mN m^−1^ min^−1^ (Figure S6, Supporting Information). In the case of a single protein, these rates can be attributed to both the charge sign and quantity for that specific protein.^[^ ^50^ ^]^ Since the orientation of water at the inner double layer results in a positive charge,^[^ ^52^, ^53^ ^]^ differences in charge can have an effect on interfacial behavior.^[^ ^50^, ^54^ ^]^ And while all crystallins are expected to be negatively‐charged at a neutral pH, it is possible that even the slight differences in surface charge of β_H_ and β_L_, with isoelectric points of 5.21 and 4.97 respectively,^[^ ^46^ ^]^ are enough to elicit differences in interfacial behavior.

Comparing mixtures of α‐crystallins, none of the combinations showed a significant change in initial rate compared to α‐crystallin alone (Figure 3a). However, when placed in combination with β_L_ or γ‐crystallin, there was an elevated final surface pressure with respect to α‐crystallin alone, suggesting more repulsive interactions between these crystallin subtypes (Figure 3a, inset). An increased initial rate and final surface pressure were also observed in the β_H_:β_L_ mixture when compared to β_H_ alone (Figure 3b). Although the final surface pressure of the α:β_H_ mixture was not statistically different when compared to β_H_ alone, there was an increased initial rate of change, 3.20 mN m^−1^ compared to 1.32 mN m^−1^, that suggested repulsive interactions (Figure 3b). When comparing mixtures to β_L_ alone, all interactions exhibited higher final surface pressure values, indicating predominately repulsive interactions (Figure 3c). From our experiments, only one notable attractive interaction was observed within the β_H_:γ mixture, which produced a lower final surface pressure and lower initial rate compared to γ‐crystallin alone (Figure 3d).

**Figure 4:**
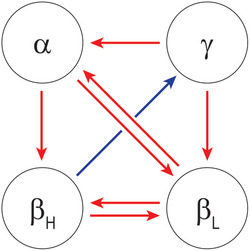
Crystallin interactions based upon surface pressure measurements. Red arrows denote repulsive interactions, while the blue arrow represents an attractive interaction. Directionality of the arrows signifies how the force is exerted, where double arrows signify a mutual repulsive interaction.

When these results were collated, several chemical interactions within the network were observed (**Figure** **4**). First, repulsive interactions were prominent within γ‐crystallins mixtures containing α or β_L_ (denoted by red arrows) but not with β_H_. This was surprising considering γ‐crystallins had been reported to exhibit overall attractive forces, especially towards α‐crystallin.^[^ ^26^, ^55^, ^56^, ^57^, ^58^ ^]^ Likewise, β‐crystallins were expected to exhibit repulsive effects in all combinations; however, only β_L_ had repulsive interactions with the other two subtypes, which is an expected feature of systems with short‐range order. β_H_ exhibited both repulsive and attractive forces (blue arrow) based on the subtype pairing (Figure 4). Since β_H_:γ crystallin was the only observed attractive interaction from our experiments, it is possible that β_H_ is responsible for coordinating γ‐crystallin in the dense milieu of the lens. Based on these correlations, we can refine our understanding of the role of β‐crystallins as structures which promote stable interfaces within the lens microstructure through specific attractive and repulsive interactions, where not all β‐crystallins should be categorized the same. Although our experimental setup is at concentrations far lower than that expected in the lens cytoplasm, the effect of protein concentration of surface pressure should only be related to the rate and magnitude at which surface tension is affected. We assume that crystallins do not undergo conformational changes at high densities that alter their surface electrostatics, so we expect these interactions to be relevant at concentrations higher than 1 mg mL^−1^.

### 2.4 In Situ Turbidity of a Crowded Hydrogel Lens Model

**Figure 5:**
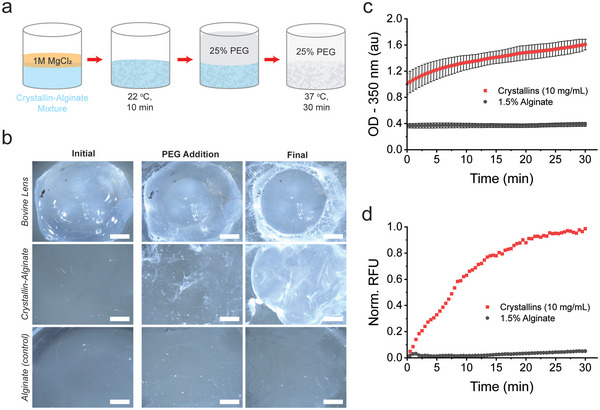
Crystallin‐alginate hydrogels exhibit turbidity in the presence of crowding agents. a) Crystallin‐alginate mixtures are polymerized and then incubated with high‐molecular‐weight PEG at physiological temperature. b) Assay was scaled up to image with a stereoscope, along with a bovine lens for comparison. Crystallin‐alginate gel shows turbidity and deswelling while the alginate control shows only some deswelling with wrinkling. Scale bars represent 2 mm. c) Kinetics were monitored spectrophotometrically over 30 min, immediately after introducing PEG d) Separate crystallin‐alginate hydrogels were monitored for protein structural changes under the same conditions using SYPRO orange to detect hydrophobic area. Kinetic data is average of biological replicates (*N* = 3) while the alginate control is an average of technical triplicates (*N* = 1). Error bars represent one standard deviation.

Although crystallins constitute a majority of the total protein content in the lens, cytoskeletal proteins comprise another vital element.^[^ ^2^, ^59^, ^60^, ^61^ ^]^ In addition to actin, the lens is rich in the structural proteins myosin, spectrin, vimentin, α‐actinin, and microtubules. There are also the unique beaded filament proteins filensin (a.k.a., BFSP1 or CP115) and phakinin (a.k.a. BFSP2 or CP49) that co‐polymerize and interact with α‐crystallin, contributing to osmotic microcirculation, accommodation, and tissue transparency.^[^ ^62^, ^63^, ^64^, ^65^, ^66^, ^67^ ^]^ Although disruption of the cytoskeletal proteins alone does not induce crystallin condensation, they do contribute to the optical quality and reduce light scattering. Based on this evidence, we speculated that a structural matrix may be a necessary element to complement our understanding of crystallin interactions within the lens cytosol. We employed a hydrogel matrix containing the anionic polysaccharide alginate to interrogate the effects of surrounding structures on the crowding and diffusion of crystallins. Crystallin‐alginate mixtures were prepared by first casting a clarified lens lysate mixture in unpolymerized alginate (1.5% w/v) then introducing 1 m MgCl_2_ to crosslink the hydrogel network (method is illustrated in **Figure** **5**a). We measured the initial turbidity of both alginate and crystallin‐alginate hydrogels after polymerization with 1 m MgCl_2_ and filled the rest of the well volume with 1x TBS (Figure S7, Supporting Information). We noticed a significant increase in light scattering of a crystallin‐alginate hydrogel with an initial optical density (OD) of 0.62 ± 0.17 compared to 0.33 ± 0.12 for alginate alone at 350 nm. However, the hydrogels were still visibly transparent and therefore we attributed this difference to the expected increased refractive index of the material when 10 mg mL^−1^ crystallins are present.

To induce crystallin turbidity within the alginate matrix, we next added 25% PEG to the hydrogel (final diluted concentration ≈8%), where each gel was then imaged and assayed spectrophotometrically (Figure 5b–d). Visually, we observed turbidity within the crystallin‐alginate hydrogel, while the alginate control remained transparent despite both hydrogels wrinkling in the presence of PEG (Figure 5b). As a comparison, we assayed the same conditions with an isolated calf lens and observed similar visual changes in opacity compared to the crystallin‐alginate mixture, especially around the edges of the lens (Figure 5b). These qualitive observations indicated that the crystallin‐alginate hydrogel was a reasonable proxy to investigate the effects of crowding in a lens‐like system. When these changes were quantified spectrophotometrically, light scattering for the crystallin‐alginate gel increased by 158% on average compared to the initial turbidity (Figure 5c; Figure S7, Supporting Information).

It should be noted that the final Mg^2+^ concentration in this assay was ≈44 mm. The concentration in a lens is expected to be closer to 5 mm.^[^ ^68^, ^69^ ^]^ However, this is not expected to significantly affect the structure or network interactions of lens crystallins, but could possibly affect the chaperone efficacy of α‐crystallin.^[^ ^46^, ^70^ ^]^ Because turbidity onset occurred almost immediately after deposition of the PEG, it was difficult to capture a nucleation or lag phase in the kinetics; however, we did observe an increased slope, approaching a plateau which is common among other aggregation mechanisms.^[^ ^71^, ^72^, ^73^ ^]^ To investigate whether this turbidity could be reversed when the buffer was exchanged from PEG to 1x TBS containing EDTA to disrupt the alginate matrix, we observed no changes in turbidity, suggesting crystallin condensation was irreversible within the matrix (Figure S8, Supporting Information).

Another consideration is whether the lens crystallins are confined within the hydrogel matrix. Alginate matrices have pore sizes of ≈5 nm.^[^ ^74^ ^]^ This is small enough that diffusion of both α‐ and β‐crystallins would be limited, but γ‐crystallin monomers would be small enough to possibly diffuse through the matrix. Since our previous results demonstrate the relationships between α‐ and β‐crystallin to be of greater importance to microstructure and turbidity of the system, it may be inconsequential that γ‐crystallin is less restricted.

To determine if the opacities were a result of changes in protein structure or aggregation in the gel matrix, we incorporated known fluorescent dyes SYPRO Orange and Thioflavin T (ThT) prior to incubation with PEG and monitored changes in turbidity of the crystallin‐alginate hydrogels (or alginate alone). We observed a 58% increase in SYPRO fluorescence over 30 min, indicating protein unfolding and an increase in solvent‐exposed area around hydrophobic residues^[^ ^75^, ^76^ ^]^ as a result of PEG crowding (Figure 5d). When this assay was repeated using ThT to monitor amyloid formation, a known marker in cataracts,^[^ ^77^, ^78^, ^79^ ^]^ we observed negligible changes in fluorescence over 30 min. This suggests that the opacity changes observed under these conditions were not a result of amyloid formation (Figure S9, Supporting Information). It is possible, however, that these changes in turbidity are associated with amorphous aggregation, given the correlation between with the rise in optical density with the SYPRO fluorescence signal changes. Future work will focus on characterization of potential formed aggregates in this system to gain mechanistic insight. Although we could not confirm whether the optical changes were a result of aggregation or liquid‐liquid phase separation, there was a good correlation between the timing of protein unfolding and the loss of transparency within our hydrogel system. Our interpretation of these results is that the crowding agent exerts pressure to disrupt the short‐range ordering of crystallins with protein unfolding occurring simultaneously or as a secondary event, possibly without aggregation. Our findings support the recent work by Schmid et al.^[^ ^80^ ^]^ which demonstrated reduced transparency in an aged mouse lens without increased insoluble protein aggregates. Based on these similarities, it is possible that our crystallin‐alginate hydrogels may serve as a simplified model to the more complex in vivo lens, but future work will be focused on refining it for mechanistic studies.

### 2.5 Delaying Crystallin‐Alginate Turbidity In Vitro

**Figure 6:**
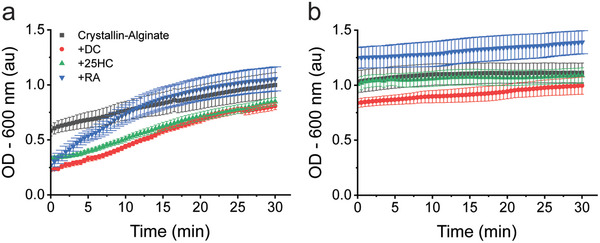
Evaluating the efficacy of anti‐cataract therapeutic candidates in our crystallin‐alginate hydrogel. A) Solutions of doxycycline (DC), 25 hydroxycholesterol (25HC), and rosmarinic acid (RA) were added after hydrogel polymerization but prior to PEG addition. PEG was added to the assay, diluting the final concentration of therapeutic to 200 µm, and turbidity kinetics were monitored at 37 °C. Data are averaged of biological replicates (*N* = 3) with error bars reporting standard error.

To further explore this possibility, we next asked whether we could alter the onset of turbidity using potential therapeutic agents. While there are currently no existing, FDA‐approved pharmaceutical treatments for prevention or reversal of age‐related cataracts, recent literature has suggested a few compounds are capable of reversing cataracts in in vitro, ex vivo, and in vivo.^[^ ^81^, ^82^, ^83^, ^84^ ^]^ However, there are conflicting results on the efficacy of these compounds.^[^ ^85^, ^86^ ^]^ We tested doxycycline (DC), 25‐hydroxycholesetrol (25HC), and Rosmarinic Acid (RA), which have all been tested on sonicated ex vivo cataracts with varying results.^[^ ^83^ ^]^ For our approach, we polymerized crystallin‐alginate hydrogels and incubated either DC, 25HC, or RA with the hydrogel for 5 min. Afterwards, a 25% PEG solution was added, and turbidity kinetics were monitored at 37 °C at 600 nm (**Figure** **6**a). The 600 nm wavelength was selected here, as both DC and RA have high baseline absorbance at 350 nm. In our assay, the average initial OD of a crystallin‐alginate hydrogel at 600 nm was 0.39, and the half‐max (OD_1/2_) based off of that was calculated to be 0.69 at 6.5 min. In the presence of RA, this was delayed to an OD_1/2_ of 9 min and reached a similar final turbidity as the control ≈1.05 AU (Figure 6a). Both DC and 25HC presented a lowered OD at every time point compared to the control, taking 20.5 and 19.0 min respectively to reach the OD_1/2_ of the control (0.69). When the therapeutic was instead added after PEG crowding, we did not observe any changes in turbidity, suggesting that once crowding has occurred, the turbidity would not reverse under these conditions (Figure 6b). This is in contrast to the results seen with ex vivo cataracts, where RA was able to restore transparency at 340 nm.^[^ ^83^ ^]^ This difference may be due to the hydrogel matrix itself, which likely limits the diffusion of crystallins and aggregates within the crowded gel. When taken together, our data reveal that the addition of these compounds can delay the onset of turbidity but cannot reverse it in our in vitro model for a crystallin rich lens.

## 3 Conclusion

While others have looked at the effects of crowding on crystallin function,^[^ ^28^, ^41^, ^87^, ^88^ ^]^ few have investigated multiple crystallins simultaneously in vitro. Our materials platform highlights the need to consider all combinations of crystallin subtypes when assaying the dense fluid‐like packing and short‐range order of the lens. We showed that protein hydrodynamic radius is correlated to solubility under crowded conditions whereby smaller proteins are more soluble but do not necessarily enhance the stability of the solution. Our results have also highlighted differences in behavior between β‐crystallin oligomers in vitro, where smaller oligomers (β_L_) exhibit better solubility under crowded conditions and are repulsive with all neighboring crystallins, including larger β‐crystallins. The larger oligomers (β_H_) produced the greatest effect at increasing the solubility of α‐crystallin under crowded conditions and exhibited attractive interactions towards γ‐crystallin. These data support different roles for β_H_ and β_L_ in lens microstructure, and by extension tissue transparency, which involves balancing attractive and repulsive forces as well as their physical size and mass ratio to the other crystallin subtypes. Therefore, the polydispersity of β‐crystallin could be critical to the organization of crystallins in the lens, as others have posed previously.^[^ ^89^ ^]^ The coordination of γ‐crystallin by β_H_ may also be an indirect effect of β_H_ on gradient light refraction. The ratios explored in these experiments highlight only a few of the possibilities that can be explored. Crystallins do not exist at 1:1 ratios in vivo and altering these in future studies could yield insight into the more granular details behind crystallin organization.

The post‐translational modification of crystallins through ageing, which begins before birth,^[^ ^90^ ^]^ may also promote some of these network interactions while further modification, or loss of specific modifications, may be disrupting the crystallin interactions that mediate transparency. This hypothesis has been described recently as “cataractogenic load”.^[^ ^2^ ^]^ Our results support that β‐crystallins are a critical aspect of the crystallin network, therefore we suggest that age‐related modifications of β‐crystallins should be investigated more closely in future discussions. For now, the lessons gleaned from our in vitro analysis provide an important breakthrough in modeling crowding of a lens. Because even changes of a few nanometers in the lens microstructure are enough to produce opacities in situ,^[^ ^41^ ^]^ it is important to develop models that allow us to simulate crystallin microstructure and protein condensation with matching changes in opacities. One approach is to embed crystallins within a material matrix, where we show that when cross‐linked in a hydrogel and crowded with PEG, the crystallins produce turbid environments reminiscent of a lens cataract. We demonstrate the ability to delay turbidity in the presence of potential cataract therapeutics at concentrations previously validated in animal models and with ex vivo human samples, supporting the potential of our platform to mimic that of a vertebrate lens.

While more work is needed to validate the full potential of this system, our method is incredibly accessible, cost‐effective, and time‐efficient. This is valuable in both academic sectors looking to explore fundamental questions of lens physiology, as well as the pharmaceutical industry for drug discovery and development. Still, there is room for improvement to resemble the lens cytoplasm more closely. A limitation of our study is that it is a highly heterogeneous biological sample, where the composition is not fully understood, and thus poses challenges for determining exact mechanisms. Future work should be focused on exploring the effects of specific post‐translational modifications and/or specific crystallin interactions on the isoform level that are producing these effects, including secondary effects associated withcritical metabolites. While further validation is needed, our results provide a proof‐of‐concept experiment that could inform future strategies toward preventing age‐related cataracts and worldwide blindness. To fully reach this potential, further work must be done to investigate mechanisms underlying specific protein‐hydrogel interactions including but not limited to studying the effect of ionic cross‐linking density and hydrogel composition or structure on crystallin turbidity of both native and combinations of recombinantly expressed protein.

## 4 Experimental Section

### Lens Extraction & Crystallin Purification

Lenses were extracted from bovine calf eyes (< 2 y), obtained locally (Research 87 Inc., Boylston, MA). Tissue was frozen whole at −20 °C and later thawed and lysed within 6 months of extraction. Thawed tissue was homogenized in a glass tissue‐grinder using PBS, pH 7.3, with 0.2% sodium azide (Fisher Bioreagents), 5 mm EDTA (Thermo Scientific), and 1 Pierce™ Protease Inhibitor Mini Tablet, EDTA‐Free (Thermo Scientific) as a lysis buffer. Lysates were centrifuged at 25 000 × g and 4 °C for 25 min. The supernatant containing soluble protein was loaded onto a Superdex 200 increase 10/300 GL size‐exclusion column (SEC; Cytiva) attached to an Äkta™ go fast performance liquid chromatography (FPLC) protein purification system (Cytiva). Material was separated and eluted with phosphate‐buffered saline (PBS) adjusted to pH 7.3 using either HCl or NaOH and vacuum‐filtered using a 0.22 mm polyvinylidene fluoride (PVDF) membrane. Chromatography was performed at 0.35 mL min^−1^ in a refrigerator (4 °C). Fractions were collected by an F9‐T fraction collector (Cytiva) in borosilicate glass tubes (Fisherbrand) and kept at 4 °C. Crystallins were repeatedly purified by SEC, batched together based on subtype (α, β_H_, β_L_, γ), and individually concentrated via centrifugation at 4000 x g for 30 min using 10 000 molecular weight cutoff filtration units (Millipore Sigma). Protein concentrations were determined by DC Assay (BioRad) to quantify dilutions of crystallins against a standard curve of bovine serum albumin (BSA). All collected samples were used or analyzed within a week after initial thawing.

### PEG Solubility Assays

A stock solution of polyethylene glycol (PEG) at 35 000 average molecular weight (Millipore; stabilized with 2‐tert‐butyl‐4‐methoxyphenol) was made by solubilizing 25% (w/v) in Milli‐Q filtered ultrapure water (Millipore Sigma; 18.2 Ω) and heated at 60 °C with occasional stirring until completely dissolved. Protein samples were prepared at 1 mg mL^−1^ total protein concentration with PEG, 10x PBS (final diluted concentration of 1x), pH 7.3, and ultrapure water. The amount of PEG ranged from 0–20% (w/v), which varied slightly at higher percentages (> 15%) to account for lower crystallin batch concentrations. Samples were mixed in 1.5 mL microcentrifuge tubes and incubated at 37 °C for 30 min prior to measurements. After incubation, samples were mixed gently by stirring with a pipette tip, then 300 µL (pathlength of ≈1 cm) were plated in triplicate onto a low‐UV absorbing 96‐well plate (Corning). The optical density (OD) of each well was measured for light scattering at 350 nm as a measure of sample turbidity at 37 °C in a spectrophotometer (Molecular Devices, SpectraMax M5).

### Hard‐Sphere Crowding Simulations

Using the open‐source software, DynamO,^[^ ^91^ ^]^ event‐driven molecular dynamics simulations (EDMD) were performed on 125 000 inelastic hard spheres of different diameters representing α‐, β‐, and γ‐crystallin. The mass and diameter of each particle were calculated from dynamic light scattering data (see Figure S2, Supporting Information) and protein ratios based on the average lens composition,^[^ ^46^ ^]^ simulated at ≈250 mg mL^−1^ which correlates to the lower cytosolic concentrations in lens fibers.^[^ ^3^ ^]^ As DynamO uses dimensionless units, the natural unit of length is the particle diameter, *σ*, and the natural unit of mass is the mass of a single particle, *m*. Both *σ* and *m* are unitary (i.e., *σ* = 1 and *m* = 1) to reduce our system into dimensionless units, where *σ* and *m* represent the diameter and mass of the largest particle, α‐crystallin, of 18.6 nm and 1.13 × 10^−18^ g, respectively. Therefore, a β‐crystallin particle had a diameter of 0.63*σ* and a mass of 0.26*m* (11.7 nm and 2.99 × 10^−19^ g) and a γ‐crystallin particle had a diameter of 0.19*σ* and 0.03*m* (3.56 nm and 3.49 × 10^−20^ g). Correspondingly, the mole fraction of α:β:γ was found to be 0.05:0.20:0.75, respectively. Fully periodic boundary conditions were imposed, and each system was compressed to packing fractions of 0.1, 0.2, 0.3, and 0.4. The temperature was set to *T* = 1 using the Andersen thermostat^[^ ^92^ ^]^ and each system was equilibrated to this temperature with sufficient collisions before any statistical analyses were performed. The radial distribution functions were sampled using the functions within DynamO^[^ ^91^ ^]^ and the static structure factors were determined using the *freud* ^[^ ^93^ ^]^ Python package and in‐house post‐processing Python scripts. All trajectories were visualized with the Visual Molecular Dynamics (VMD) software.^[^ ^94^ ^]^

### Tensiometry

Measurements were carried out on an Attension Theta pulsating drop tensiometer (Biolin Scientific) to monitor the surface tension of each droplet with respect to time. A Hamilton glass syringe connected to a peristaltic pump and inlet line was used to load protein solutions at 1 mg mL^−1^. A 21 µL droplet was expelled from the tip of a blunt, 33‐gauge stainless steel needle. Droplets were imaged over 40 min at 0.11 fps, and the shape of the droplet was fit with the Young‐Laplace equation to calculate surface tension at each time point. Three drops were individually analyzed for each sample; this was repeated for a biological replicate (*N = 2*). Between experiments, the syringe was rinsed with 100% ethyl alcohol (Thermo Scientific), then ultrapure water. All measurements were performed at room temperature (21–24 °C) with a relative humidity of 35±11%.

### Stereoscope Imaging of Lens and Crystallin‐Alginate Crowding

Lens lysates were prepared as described using superspeed centrifugation (25 000 × g and 4 °C for 25 min), and the resulting supernatant was then centrifuged again at 4000 x g for 20 min using 10 000 molecular weight cutoff filtration units (Millipore Sigma) to separate smaller, non‐crystallin proteins and peptides. Lastly, solutions were vacuum filtered through a 0.22 µm polyvinylidene fluoride membrane (Millipore Sigma) to remove any pre‐formed aggregates. Total protein concentrations of diluted lysates were determined by DC Assay (BioRad) against a standard curve of bovine serum albumin (BSA). Sodium Alginate (MP Biomedicals) was pre‐mixed in deionized water with constant stirring at 60 °C to a final concentration of 3% (w/v). Crystallin‐alginate mixtures were prepared at noted protein concentrations with 1.5% sodium alginate and 1x Tris‐buffered saline (TBS) in a 15 mL centrifuge tube. The TBS was prepared as a 10x stock using Tris‐HCl (Promega), sodium chloride (Fisher Bioreagents), and deionized water. Solutions of MgCl_2_ were prepared at 1 m concentration using magnesium chloride hexahydrate (Fisher Bioreagents) in deionized water. Each gel mixture was pipetted into a disposable clear‐bottom 6‐well plate (Falcon). Using a transfer bulb pipette, hydrogels were covered with 1 m MgCl_2_ and allowed to polymerize for 10 min at room temperature with gentle orbital agitation. Separately, one calf lens was thawed in 1x TBS at room temperature for 10 min. The remaining solutions of MgCl_2_ and TBS were replaced with 2 mL of 25% (w/v) PEG‐35 000 (Millipore). Live imaging was recorded using a light stereoscope (Nikon SMZ18).

### In Vitro Crystallin Turbidity Assays in a Hydrogel Matrix

Crystallin‐Alginate hydrogels were prepared as mentioned above but at a smaller volume, along with an alginate control. For the assay, 500 µL of the crystallin‐alginate mixture was pipetted in triplicate onto a low‐UV absorbing 48‐well plate (Corning Costar). Then 100 µL of 1 m MgCl_2_ were pipetted on top of each well and incubated at room temperature for 10 min with a plate lid to polymerize the hydrogel network. Afterwards, 1x TBS was added to bring the total volume of each well to 1.5 mL. Initial light scattering was measured as OD at 350 nm in a plate‐reader spectrophotometer (SpectraMax M5; Molecular Devices) (Figure S7, Supporting Information). After measuring OD, 500 µL of solution was aspirated from each well using a micropipette and replaced with 25% (w/v) PEG‐35000 (Millipore). The plate was then incubated at 37 °C for 30 min, tracking OD at 350 nm every 30 s. This process was repeated using three lenses (*N = 3*) and the data were averaged.

### Fluorescent Assays (SYPRO Orange, Thioflavin T)

Stocks of SYPRO Orange (100x, Invitrogen) and Thioflavin T (Thioflavin T benzothiazole salt, Abcam) were prepared ahead of time in dimethyl sulfoxide (DMSO) and stored at −20 °C. Hydrogel mixtures were prepared as previously described for a 48‐well plate assay. Prior to the addition of PEG, either SYPRO Orange or Thioflavin T were added to a final concentration of 1x or 100 µm, respectively. The wells were then filled with 25% (w/v) PEG‐35 000 (Millipore) to the final volume. Fluorescence for SYPRO Orange was measured by excitation at 470 nm and emission of 570 nm with a 550 nm cutoff filter; thioflavin T fluorescence was measured by excitation at 440 nm and emission of 485 nm with a 455 nm cutoff filter. Kinetics were measured at 37 °C over 30 min at 30 s intervals.

### Anti‐Cataract Therapeutic Assays

Stocks of doxycycline (DC; Fisher BioReagents), 25 hydroxycholesterol (25HC; Sigma Aldrich), and rosmarinic acid (RA; Thermo Scientific) were prepared ahead of time. RA was dissolved in 1x TBS. Both DC and 25HC were dissolved first in dimethyl sulfoxide (DMSO), then diluted with 1x TBS. All compounds were at a final stock concentration of 1.2 mm. Crystallin‐alginate gels were polymerized in a 48‐well plate according to the previous method. Prior to the addition of PEG, 250 µL of anti‐cataract therapeutic was added from the stock solution and incubated at room temperature for 5 min to allow the compound to start perfusing through the hydrogel. Then each well was filled with 25% PEG to a final volume of 1.5 mL. Turbidity was tracked as OD‐350 nm and OD‐600 nm for 30 min at 37 °C in a spectrophotometer (Molecular Devices, SpectraMax M5).

### Statistical Analyses

PEG solubility assays were repeated for biological replicates (*N = 3*) and the data were averaged with error bars reporting one standard deviation (σ). Data were fitted to the Boltzmann equation using Origin Pro software. Adjusted R^2^ values ranged between 0.969‐0.999 apart from γ‐crystallin (0.440), which was not analyzed for ½ solubility point. Although the β_L_:γ mixture had a good fit (adjusted R^2^ = 0.999), it is unclear whether 20% PEG was high enough to reach a final turbidity, therefore we could not confidently report the ½ solubility.

Surface tension data was converted to surface pressure (Π) by subtracting the surface tension at each timepoint from the initial measurement at *t_0_*. Surface pressure data sets were analyzed for initial rate and final surface pressure. Initial rate was calculated by taking the slope at each point for the first 100 seconds where the graph is linear. Data points were averaged for individual data sets (*n* = 72) with error bars reporting standard error of the mean (S.E.M.). Final achieved surface pressure was calculated by averaging the last 240 s, i.e., the last 10%, of each trial. These values were also averaged (*n* = 162) with error bars reporting standard error of the mean. One‐way ANOVA statistical testing was carried out using Microsoft Excel with a significance threshold of 0.05. Resulting p‐values for initial rate and average surface pressure were 1.58E‐4 and 2.01E‐89, respectively. Post‐hoc analysis was done by a series of two‐tailed, homoscedastic t‐tests using a Bonferroni correction to determine significance.

Turbidity assays performed with spectrophotometry were repeated in biological triplicates (*N = 3*) and the data were averaged with error bars reporting one standard deviation (σ). Normalization of SYPRO fluorescent signal was done in Microsoft Excel for each individual trial by taking the data at any time point *t* and transforming it with the equation:

(1) $$\frac{\left(t-MIN\right)}{MAX-MIN}$$

where “MIN” and “MAX” are the minimum and maximum fluorescent signals recorded within that trial. For the alginate control samples, which exhibited minimal fluorescent signal, the “MAX” was instead the maximum signal achieved by the crystallin‐alginate condition in the same trial. Normalized data were averaged (*n* = 9) across three biological replicates (*N = 3*), consisting of technical triplicates.

## Conflict of Interest

The authors declare no conflict of interest.

## Author Contributions

M.R.B., J.Y., and L.F.D. originated the ideas and designed the experiments. M.R.B. and C.D. performed the PEG solubility experiments. M.R.B., S.A.H., and C.D. performed the surface tensiometry. J.Y. performed the hard‐sphere simulations and analyses. M.R.B. and S.A.H. conducted the hydrogel and lens turbidity assays. M.R.B., J.Y., and L.F.D. wrote the manuscript.

## Supporting information

Supporting Information

## Data Availability

The data that support the findings of this study are available from the corresponding author upon reasonable request.
