# Supplementary material for: Design and Characterization of Model Systems that Promote and Disrupt Transparency of Vertebrate Crystallins In Vitro
Source: Adv Sci (Weinh). 2023 Oct 28;10(35):2303279. doi: 10.1002/advs.202303279 (PMC10724405; doi:10.1002/advs.202303279)
Supplement: Supplementary file 1 — Supporting Information [file ADVS-10-2303279-s001.pdf]

ADVANCED SCIENCE
Open Access

Supporting Information

for *Adv. Sci.*, DOI 10.1002/advs.202303279

Design and Characterization of Model Systems that Promote and Disrupt Transparency of Vertebrate Crystallins In Vitro

*Michael R. Bergman, Sophia A. Hernandez, Caitlin Deffler, Jingjie Yeo and Leila F. Deravi**

Supplementary Information for

Physicochemical properties of vertebrate crystallins that promote lens transparency

Michael R. Bergman, Sophia A. Hernandez, Caitlin Deffler, Jingjie Yeo, and Leila F. Deravi*

*To whom correspondence should be addressed, Leila F. Deravi

Email: l.deravi@northeastern.edu

This PDF file includes:

Figures S1-S9

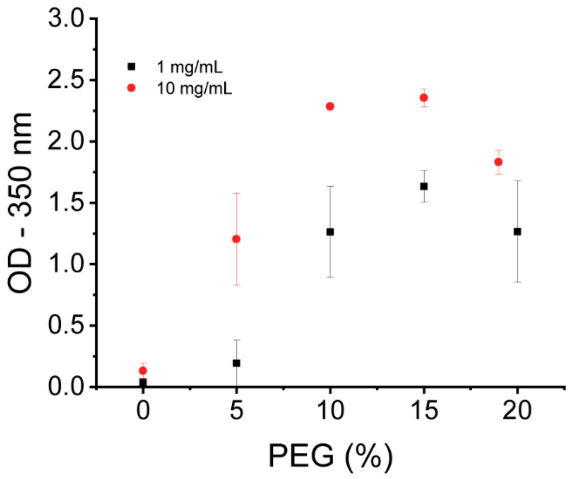

Fig. S1. Crowding agents test the solubility of crystallin mixtures (α, β, γ) from bovine lens. Crystallins were extracted and filtered from bovine lens extract, then mixed with increasing weight percentages of high-molecular-weight polyethylene glycol (PEG). This maintains the native mass ratios for α, β, γ at 35:37:21. Turbidity was measured as a function of optical density (OD) and curves were best fit to a Boltzmann equation. Results are listed in Table 1. Data points represent averages of biological replicates (*N=3*) with error bars representing one standard deviation.

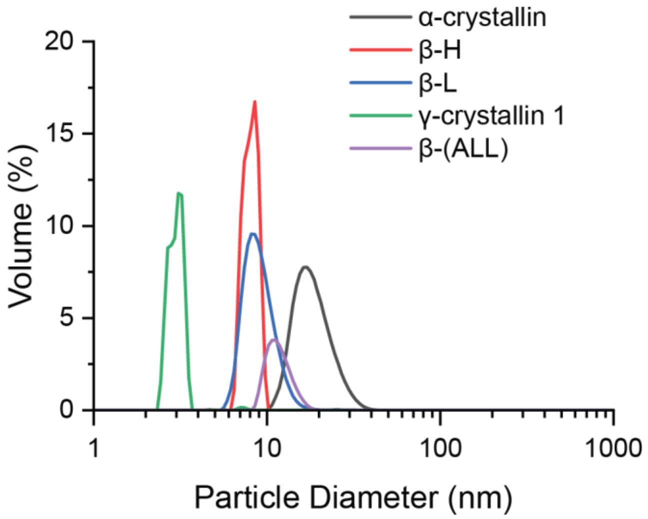

Fig. S2. Particle diameter measurements for solutions of bovine crystallins. Each population was purified using previous protocols. Samples were diluted to 0.5 mg mL^{-1} final protein concentration with PBS and measured; data are an average of three replicates. The β-(ALL) represents the mixed population of β-crystallins where they were purified using SEC-FPLC but pooled together prior to concentration determination and DLS measurements. Our DLS results had diameters of 18 nm, 12 nm, and 4 nm for α-, β-, and γ-crystallin, respectively. These are similar or slightly smaller than those previously reported for *in situ* samples.[1-4]

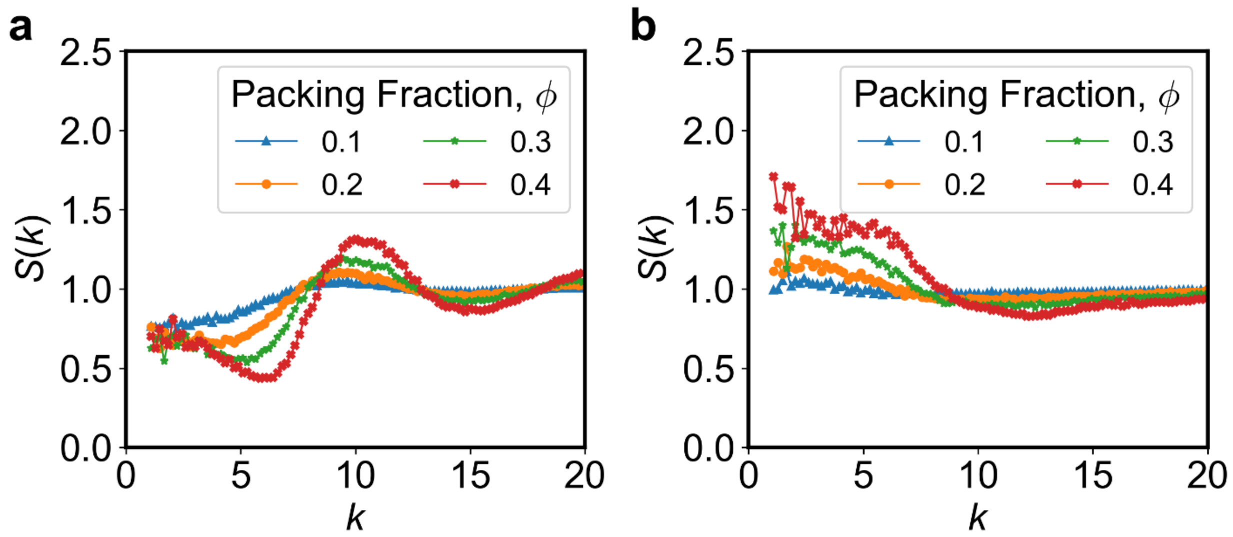

Fig. S3. Plots of the structure factor of (a) β-crystallins, and (b) γ-crystallins in the polydisperse α+β+γ-crystallin systems as the volume fraction increased from 0.1 to 0.4. The static structure factors were determined using the *freud*[5] Python package and in-house post-processing Python scripts.

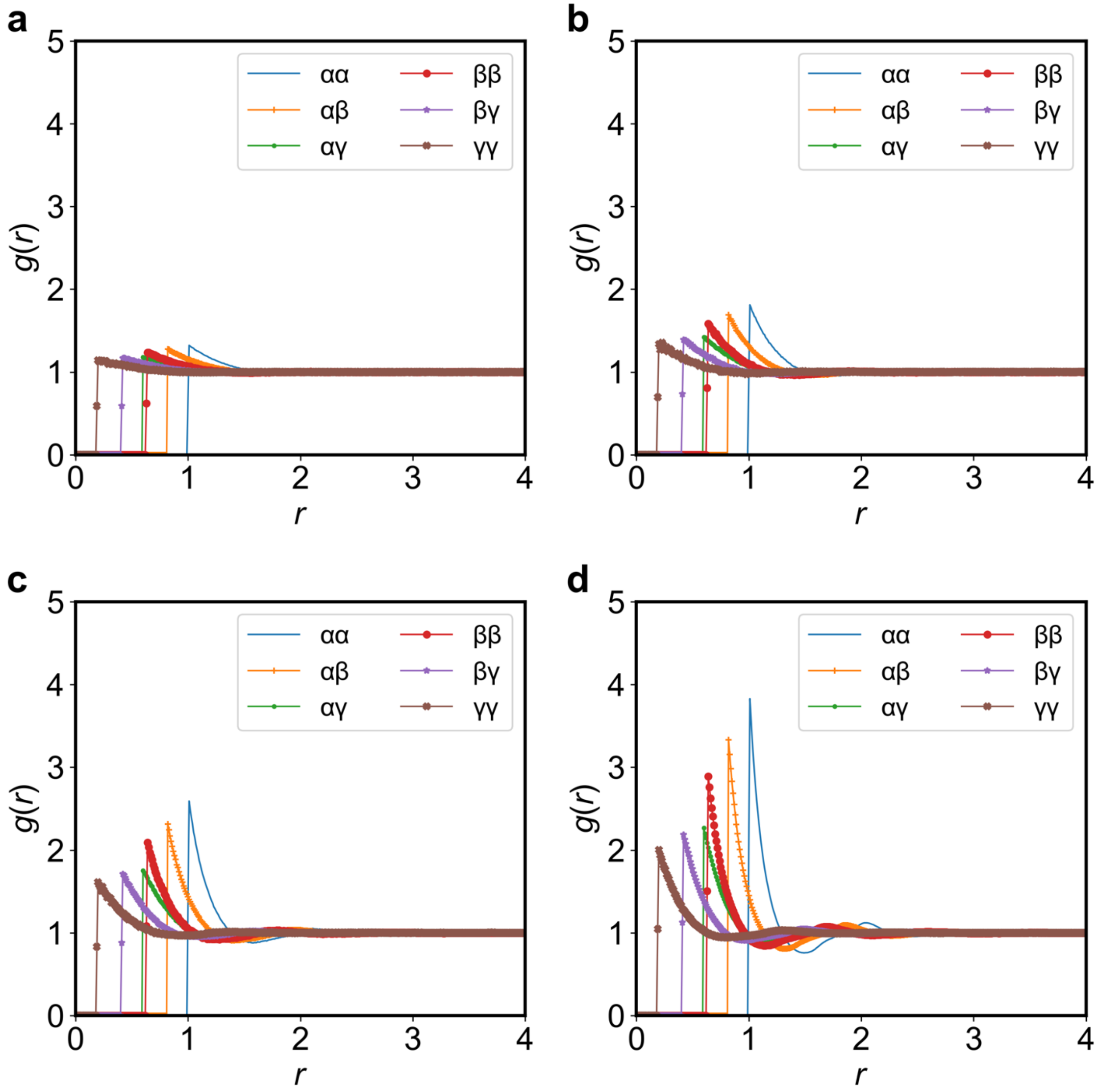

Fig. S4. Plots of the radii of gyration between every combination of crystallins in the polydisperse systems with equal numbers (1:1:1) of α, β, and γ-crystallin as the volume fraction increased from (a - d) 0.1 to 0.4. The radial distribution functions were sampled using the functions within DynamO. [6]

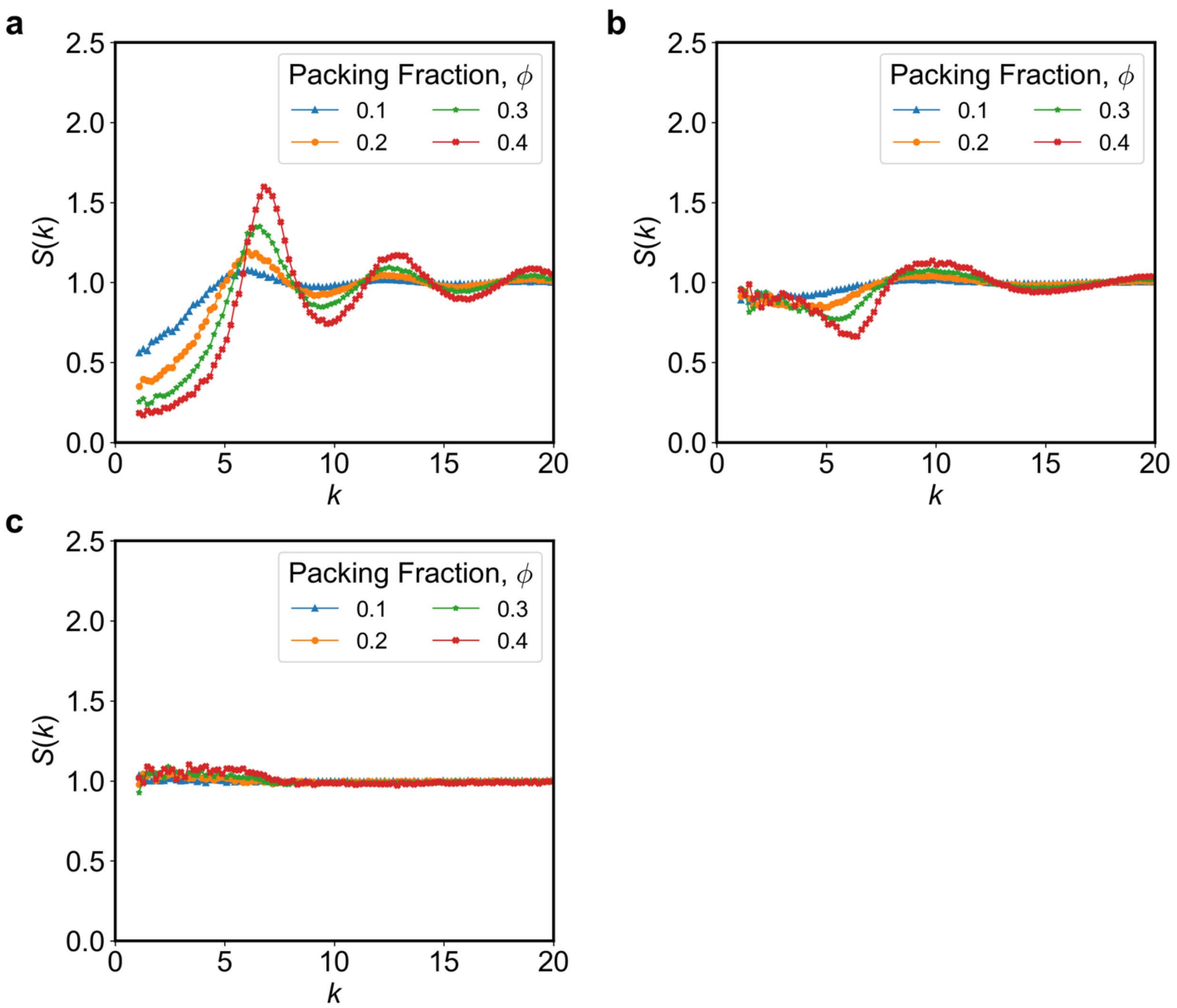

Fig. S5. Plots of the structure factor between every combination of crystallins in the polydisperse α+β+γ-crystallin systems with equal ratios (1:1:1) as the packing fraction increased from 0.1 to 0.4 for (a) α-crystallins, (b) β-crystallins, and (b) γ-crystallins.

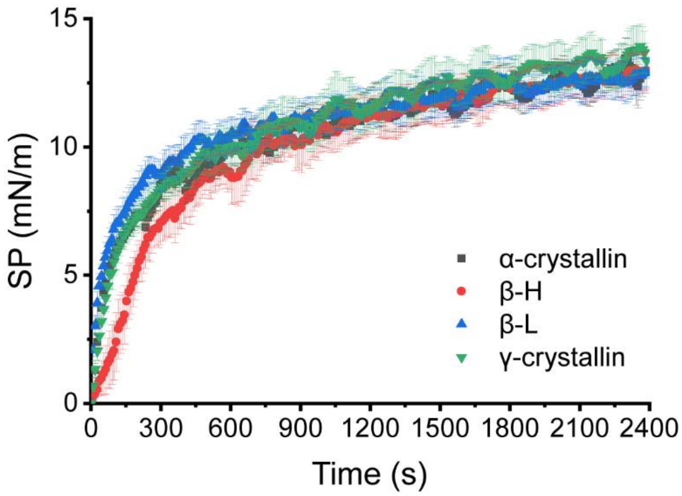

Fig. S6. Surface pressure profiles of isolated bovine lens crystallins. Data are averaged from biological replicates (*N=2*) with error bars reporting standard error of the mean.

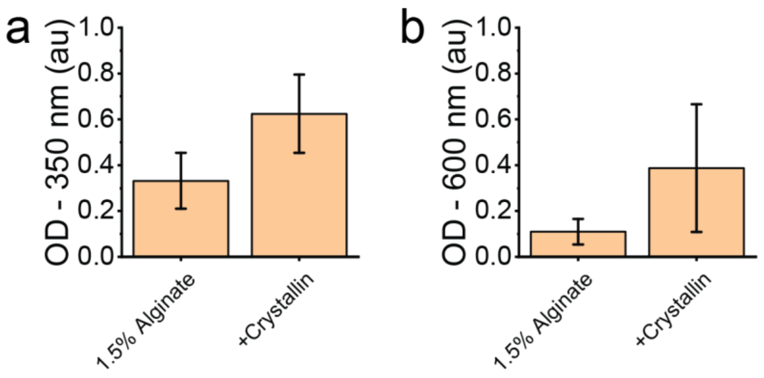

Fig. S7. Initial turbidity of hydrogels prior to PEG addition. Light scattering was measured at two wavelengths, (a) 350 and (b) 600 nm. Values are averaged of three biological replicates with error bars reporting one standard deviation.

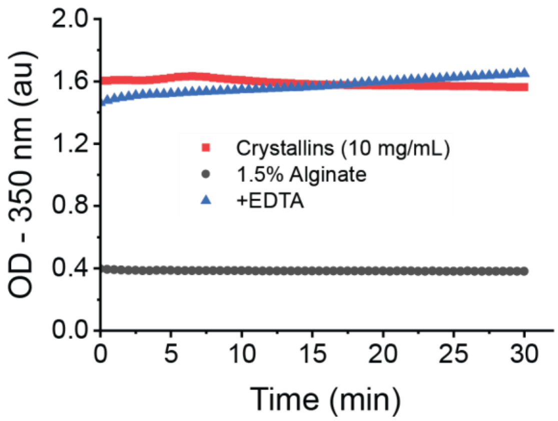

Fig. S8. Monitoring for disaggregation in alginate hydrogels. The turbidity of crystallin-alginate hydrogels was measured spectrophotometrically at 37 °C following a buffer-exchange to remove the excess PEG and replaced with 1x TBS. The addition of EDTA with the 1x TBS (blue) was used to de-polymerize the alginate matrix. Data represents an average of biological replicates (*N=3*).

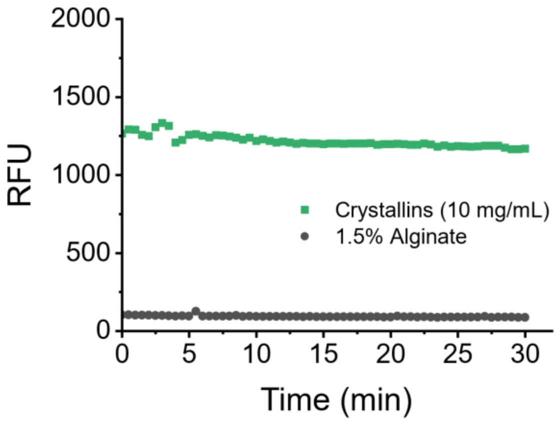

Fig. S9. PEG crowding of crystallin-alginate hydrogel in the presence of Thioflavin T. Fluorescence was measured by excitation at 440 nm and emission at 485 nm at 37 °C over the course of 30 minutes after addition of high-molecular weight PEG.

Additional SI References:

[1] A. J. Kiss, A. Y. Mirarefi, S. Ramakrishnan, C. F. Zukoski, A. L. Devries, C. H. Cheng, *J Exp Biol* **2004**, *207* (Pt 26), 4633, https://doi.org/10.1242/jeb.01312.

[2] A. Y. Mirarefi, S. Boutet, S. Ramakrishnan, A. J. Kiss, C. H. Cheng, A. L. Devries, I. K. Robinson, C. F. Zukoski, *Biochim Biophys Acta* **2010**, *1800* (6), 556, https://doi.org/10.1016/j.bbagen.2010.02.004.

[3] R. R. Ansari, K. I. Suh, S. Dunker, N. Kitaya, J. Sebag, *Exp Eye Res* **2001**, *73* (6), 859, https://doi.org/10.1006/exer.2001.1097.

[4] M. Latina, L. T. Chylack, Jr., P. Fagerholm, I. Nishio, T. Tanaka, B. M. Palmquist, *Invest Ophthalmol Vis Sci* **1987**, *28* (1), 175.

[5] V. Ramasubramani, B. D. Dice, E. S. Harper, M. P. Spellings, J. A. Anderson, S. C. Glotzer, *Computer Physics Communications* **2020**, *254*, 107275.

[6] M. N. Bannerman, R. Sargant, L. Lue, *Journal of computational chemistry* **2011**, *32* (15), 3329.
